# Effects of acute essential amino acid intake on post-prandial raw bioimpedance and fluid shifts between healthy young and older adults: An exploratory pilot study

**DOI:** 10.2478/joeb-2025-0016

**Published:** 2025-11-28

**Authors:** Callie L. Unrein, David D. Church, Arny A. Ferrando, Robert R. Wolfe, Katie R. Hirsch

**Affiliations:** Department of Exercise Science, Arnold School of Public Health, University of South Carolina, Columbia, SC, USA; Center for Translational Research in Aging & Longevity, University of Arkansas for Medical Sciences, Little Rock, AR, USA

**Keywords:** Bioimpedance, body water, essential amino acid

## Abstract

**Introduction:**

Age-related anabolic resistance can lead to reduced muscle mass but can be costly and timely to diagnose. Multi-frequency bioelectrical impedance analysis could potentially be used as a non-invasive tool for the assessment of anabolic resistance as changes in frequency-dependent impedance values may reflect fluid shifts occurring with nutrient uptake. This exploratory pilot study evaluated the effects of acute essential amino acid (EAA) intake on raw bioimpedance and fluid shifts in healthy young adults (YA, n=5) and older adults (OA, n=7).

**Methods:**

Participants completed a five-hour protocol with multifrequency bioelectrical impedance analysis (MF-BIA) at baseline and every 30 minutes post-consumption of a 10 g EAA beverage. Whole-body and segmental values for impedance (Z), resistance (R), reactance (Xc), phase angle (PhA), and body water compartments were assessed.

**Results:**

YA demonstrated significantly higher Z, R, Xc, and PhA values compared to OA (p<0.05), particularly in the leg segments and at 50 kHz. Time effects revealed declines in R (p=0.013) and Xc (p=0.002) following EAA ingestion, consistent with postprandial fluid shifts. Fluid analysis showed significant group differences only for ECW/ICW ratio (p=0.001–0.004) with OA > YA and increases in TBW, ICW, and ECW over time.

**Conclusion:**

Raw bioimpedance values distinguished between age groups and reflected acute responses to nutrient intake. These findings suggest MF-BIA may be sensitive to short-term physiological changes and, with further validation, could support assessments of muscle quality and nutritional responsiveness.

## Introduction

Aging is marked by declining muscle mass and function, partly due to anabolic resistance—the reduced capacity to synthesize muscle protein in response to nutrients and exercise (1). This age-related anabolic resistance can lead to reduced muscle mass (2), quality (3), strength (4), and increased fall risk (5). Clinical diagnosis and monitoring of muscle mass and function typically requires physical performance tests, imaging (e.g., DXA), and questionnaires (6), but assessments of anabolic resistance are clinically impractical (i.e. isotope tracers), emphasizing the need for more accessible methods.

Multifrequency bioelectrical impedance analysis (MF-BIA) offers a non-invasive, portable, and low-cost method to estimate body composition (7), in addition to capturing raw bioimpedance variables like impedance (Z), resistance (R), reactance (Xc), and phase angle (PhA). These metrics relate to cellular integrity (8), muscle health (9), and metabolic status (10). Impedance is the delay of the electrical current into cells of the body and is related to hydration of body fluids and tissues (11). Resistance is the opposition of the flow of the electrical current and is an indicator of cellular hydration (8). For example, skeletal muscle mass is higher in water and electrolyte content and has higher conductivity, resulting in a lower resistance than adipose tissue. Reactance is the delay of the electrical current by cell membranes and is a function of cell membrane integrity (8). For example, in individuals with chronic diseases such as cancer or severe malnutrition, the integrity of cell membranes is often compromised resulting in lower Xc values compared to healthy individuals (12). Lastly, PhA in particular, is a marker of muscle quality (9), nutritional status (13), and mortality risk (14). Age-related differences in raw bioimpedance have been observed (15), with prior research indicating lower PhA and Xc in individuals with sarcopenia (16).

MF-BIA also estimates body fluid compartments—total body water (TBW), extracellular water (ECW), intracellular water (ICW), and extracellular-to-intracellular water (ECW/ICW) ratio (17), which reflect hydration, muscle composition, and disease status (18). With aging, loss of skeletal muscle mass can lead to reduced TBW, mainly due to decreased ICW (19). Conversely, conditions such as edema can result in maintenance of TBW, despite reductions in skeletal muscle mass, due to increased ECW and ECW/ICW (20,21). Tracking these shifts with MF-BIA could help clarify and quantify age-related physiological changes between fluid compartments.

While single timepoint MF-BIA provides descriptive insights, repeated measurements post-feeding may reveal dynamic nutrient responses, particularly differences in fluid shifts between healthy individuals and those with sarcopenia. In healthy muscle, EAA uptake would shift fluid intracellularly, decreasing intracellular R and increasing intracellular Xc and PhA (22). With anabolic resistance, this intracellular uptake would be blunted, reflected in smaller changes in raw bioimpedance. As such, tracking changes in raw bioimpedance outcomes over a postprandial period may offer a novel way to detect or evaluate muscle responsiveness to nutrition. Therefore, the purpose of this exploratory study was to evaluate the effects of an acute EAA challenge on raw bioimpedance and fluid shifts in healthy young adults (YA) and older adults (OA).

## Materials and methods

### Participants

A sub-sample of 12 participants (four males and eight females) from a larger metabolic study, who were willing and able to complete the extra measurements, were included in this exploratory analysis (NCT05117112). The larger metabolic study sought to characterize muscle health by evaluating skeletal muscle kinetics to an EAA challenge. Participants were separated into two separate groups by age. These groups included: healthy young adults (YA: n=5; Age: 18–30 years) and older adults (OA: n=7; Age: 70–89 years). Participants were excluded if they had a reported history of metabolic or endocrine disorder (e.g., diabetes, polycystic ovarian syndrome), chemotherapy or radiation therapy within the six months prior to enrollment, history of gastrointestinal surgery (e.g., lap band, gastric sleeve), current use of corticosteroids, testosterone, IGF-1, or use of similar anabolic agents, and those who were pregnant, planning to become pregnant, or currently breastfeeding. All participants provided written informed consent prior to participation. Participant demographics are presented in Table 1.

**Table 1.** Participant demographics

	**YA**	**OA**
**12 (4M/8F)**	5 (1M/4F)	7 (3M/4F)
**Age (yrs)**	27.4 ± 1.3	75.1 ± 0.9*
**Height (cm)**	165.2 ± 2.6	168.4 ± 2.7
**Weight (kg)**	75.4 ± 10.5	79.6 ± 3.3
**BMI (kg/m^2^)**	27.6 ± 3.7	28.1 ± 1.0
**SMM (kg)**	26.2 ± 1.8	27.5 ± 1.5
**%BF**	33.8 ± 7.5	36.5 ± 2.3
**SMM of Right Leg**	7.2 ± 1.1	7.9 ± 1.1*
**FM of Right Leg**	3.9 ± 2.5	4.0 ± 0.7

YA: young adult; OA: older adult;

* *Statistically different from YA (p<0.001).*

### Experimental design

**Fig.1:**
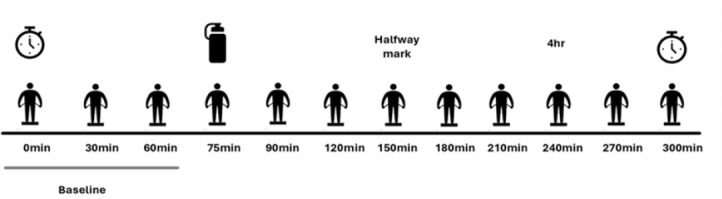
Experimental design. Timers indicate the start and stop of the study protocol. The bottle indicates when the EAA drink was consumed. The silhouettes refer to each MF-BIA measurement.

Participants arrived at the lab following an 8-hour fast, having abstained from caffeine for 12 hours, and vigorous exercise for the past 24 hours. The five-hour study protocol began with three baseline MF-BIA measurements (InBody 770, Seoul, South Korea) taken over the course of one hour, at 0, 30, and 60 minutes, respectively. At the 75th minute, participants consumed an essential amino acid (EAA) drink containing 10 grams of free-form EAAs, which they were instructed to finish within one minute. Following EAA consumption, postprandial MF-BIA measurements began again at the 90th minute and continued every 30 minutes for the subsequent four hours. Participants remined fasted and rested in a semi-reclined position for the duration of the protocol, standing or moving around only to use the restroom or prior to completing MF-BIA tests. An overview of the study protocol is represented in Figure 1.

### Multifrequency bioelectrical impedance analysis

Whole-body and segmental Z, R, Xc, and PhA were assessed using an upright MF-BIA device (InBody 770; Biospace Co., Seoul, South Korea) following manufacturer guidelines (Hirsch et al., 2023). The device utilizes six frequencies (1, 5, 50, 250, 500, 1000 kHz) to measure segmental Z (right arm, left arm, trunk, right leg, left leg). Segmental Xc is automatically provided by the device at 5, 50, and 250 kHz. Whole-body PhA at 50 kHz is also automatically provided by the device. The remaining whole-body and segmental raw bioimpedance values for R and PhA at 5, 50, and 250 kHz were manually calculated using the equations described below. The remaining bioimpedance values at 1, 500, and 1000 kHz could not be calculated from the available data. Therefore, whole-body and segmental bioimpedance measures of Z, R, Xc, and PhA at 5, 50, and 250 kHz were used for analysis. For fluid shifts, whole-body and segmental TBW, ECW and ICW were measured at 50 kHz and automatically provided by the MF-BIA device. Whole-body and segmental ECW/ICW ratio was manually calculated by dividing the ECW values by the ICW values provided.

### Calculations

Bioimpedance values at 5, 50, and 250 kHz were calculated using equations 1 and 2 and ECW/ICW ratio was calculated at 50 kHz using equation 3 as described below:

Measurements for R were manually calculated using equation 1 from Z and Xc measures automatically provided by the MF-BIA device.

Equation 1: $$R=\sqrt{z^{2}-Xc^{2}}$$

Measurements of PhA were manually calculated using equation 2 from Xc automatically provided by the MF-BIA device and the manually calculated R values.

Equation 2: $$\mathrm{PhA}=\arctan\left(\frac{\mathrm{xc}}{R}\right)\cdot \frac{180{}^{\circ}}{\pi }$$

Measurements for ECW/ICW ratio were manually calculated by dividing ECW by ICW using equation 3.

Equation 3: $$\frac{\mathrm{ECW}}{\mathrm{ICW}}\mathrm{ratio}=\mathrm{ECW}÷\mathrm{ICW}$$

### EAA beverage

The EAA beverage contained 10 g of free-form EAA (total of 17.8 grams of commercial product; XS Muscle Multiplier; Amway; Ada Michigan) dissolved into approximately 10 ounces of water. One serving of the powder contained 4.1 g of a proprietary amino acid blend of L-Leucine, L-Valine, L-Isoleucine, L-Lysine HCI, L-Arginine HCl, L-Threonine, L-Phenylalanine, L-Methionine, L-Histidine, and L-Tryptophan and has been shown to stimulate a significant muscle protein synthetic response (23). Each drink was prepared by the research team. Participants were instructed to consume the entire drink within one minute.

### Informed consent

Informed consent was obtained from all individuals included in this study.

### Ethical approval

The research related to human use has been complied with all relevant national regulations, institutional policies and in accordance with the tenets of the Helsinki Declaration and has been approved by the authors’ institutional review board or equivalent committee.

## Statistical analysis

In order to evaluate the effects of acute EAA intake on whole-body and segmental raw bioimpedance values (Z, R, Xc, PhA), separate 2 × 12 repeated measures one-way analysis of variance (ANOVA) (Group [YA, OA] × Time [0, 30, 60, 75, 90, 120, 150, 180, 210, 240, 270, 300]) were conducted to compare differences between young and older adult groups over time. Partial eta squared (η^2^) values were reported as measures of effect size.

In order to evaluate the effects of acute EAA intake on whole-body and segmental fluid shifts (TBW, ECW, ICW, ECW/ICW), separate 2 × 12 repeated measures ANOVAs (Group X Time) were conducted to compare differences between young and older groups over time. Partial eta squared (η^2^) values were reported as measures of effect size. Significant interaction and main effects were followed by pairwise comparisons using Tukey’s post-hoc tests.

To account for sex imbalances between the YA and OA groups, which could impact bioimpedance outcomes, a female-only analysis of whole-body bioimpedance and fluid measures was conducted using the same statistical procedures as described above (Demographics presented in Supplementary Table 1). All statistical tests were conducted using SPSS (Version 29, IBM, Armonk, NY, USA), using an α=0.05 to determine statistical significance.

## Results

### Whole-body bioimpedance

**Fig.2:**
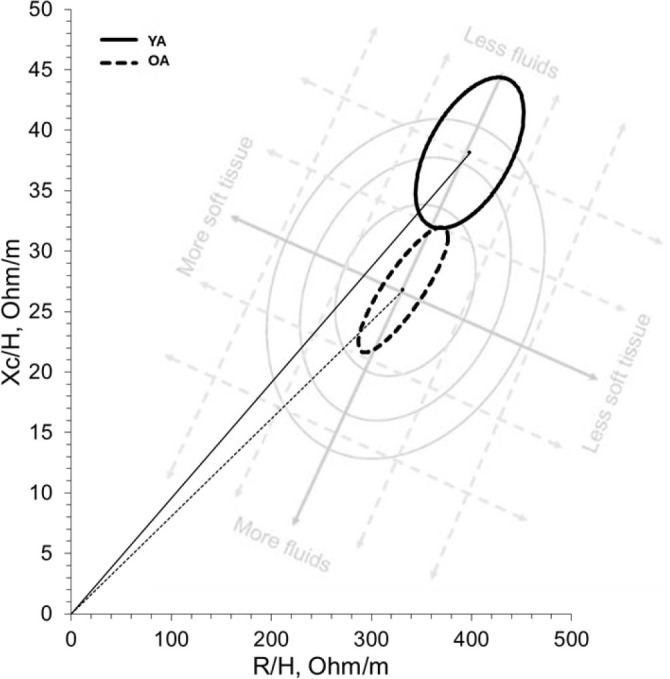
Bioelectrical impedance vector analysis between YA and OA. *Background watermark is not drawn to scale and provided solely for context.

No significant interaction effects were observed for any whole-body bioimpedance variable at any frequency (p > 0.05). However, there were significant main effects of group and time for Z, R, Xc at all frequencies (5, 50, and 250 kHz). Specifically, group effects were significant for Z (p = 0.005–0.017, η^2^ = 0.448–0.557), R (p = 0.003–0.009, η^2^ = 0.506–0.598), and Xc (p = 0.001–0.018, η^2^ = 0.447–0.715). Time effects were also significant for Z (p < 0.001, η^2^ = 0.542–0.553), R (p < 0.001, η^2^ = 0.455–0.552), and Xc (p < 0.001, η^2^ = 0.418–0.530). For PhA, a group effect was observed only at 50 kHz (p = 0.005, η^2^ = 0.557), while time effects were significant at 5 kHz (p = 0.005, η^2^ = 0.331) and 50 kHz (p = 0.017, η^2^ = 0.253), but not at 250 kHz (p > 0.05). Similar results were observed in the female only analysis between YA and OA (Supplementary Table 2). Graphical representation of whole-body bioimpedance differences between YA and OA are represented in Figure 2.

**Table 2:** Whole-body bioimpedance results at 5, 50, and 250 kHz for YA and OA.

	**Bioimpedance**	**YA**	**OA**	**Interaction Effect (p-value)**	**Group Effect (p-value)**	**Time Effect (p-value)**
**5 kHz**	Z (Ω)	741.46 ± 26.14	620.0 ± 22.09*	0.238	0.005	<0.001
	R (Ω)	740.82 ± 26.12	619.49 ± 22.08*	0.237	0.005	<0.001
	Xc (Ω)	30.8 ± 1.54	25.05 ± 1.31*	0.751	0.018	0.001
	PhA (º)	2.39 ± 0.11	2.31 ± 0.09	0.884	0.597	0.005
**50 kHz**	Z (Ω)	660.9 ± 23.73	560.47 ± 20.06*	0.241	0.009	<0.001
	R (Ω)	657.82 ± 23.65	558.62 ± 19.99*	0.241	0.009	<0.001
	Xc (Ω)	62.99 ± 2.73	45.12 ± 2.31*	0.350	0.001	<0.001
	PhA (º)	5.53 ± 0.18	4.67 ± 0.16*	0.296	0.005	0.017
**250 kHz**	Z (Ω)	594.48 ± 21.57	514.0 ± 18.23*	0.238	0.017	<0.001
	R (Ω)	704.1 ± 38.3	510.82 ± 32.37*	0.193	0.003	0.001
	Xc (Ω)	50.29 ± 2.19	36.97 ± 1.85*	0.281	0.001	<0.001
	PhA (º)	4.11 ± 0.1	4.15 ± 0.08	0.663	0.757	0.680

YA: Young adult; OA: older adult

* *Statistically different from YA (p<0.05)*

**Fig.3:**
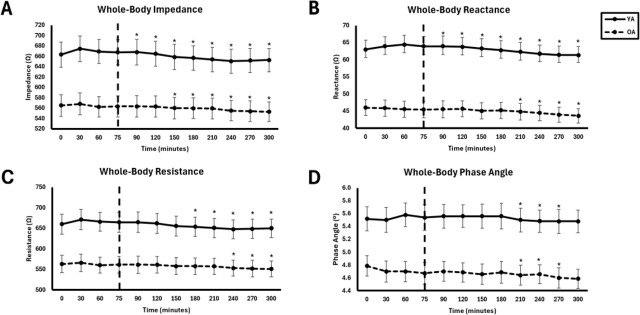
Whole-body bioimpedance at 50 kHz between young adults (YA) and older adults (OA) across time. The EAA challenge was consumed at the 75^th^ minute. *Significantly different from pre-EAA consumption timepoints (0, 30, 60, or 75 minutes; p<0.05).

General trends across frequencies: YA demonstrated higher bioimpedance values compared to OA. Group differences were most pronounced at 50 kHz, with Z and R demonstrating the highest frequency of significant effects, followed by Xc and PhA. Over time, bioimpedance values were highest at baseline and decreased over time. This effect was most pronounced at 50 kHz, with Z and R demonstrating the highest frequency of significant effects, followed by Xc and PhA. Consolidated whole-body bioimpedance results are presented in Table 2, and time-related trends are shown in Figure 3.

### Segmental bioimpedance

**Fig.4:**
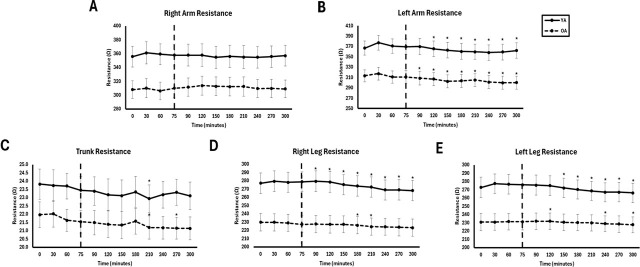
Segmental resistance at 50 kHz between young adults (YA) and older adults (OA) across time. The EAA challenge was consumed at the 75^th^ minute. *Significantly different from pre-EAA consumption timepoints (0, 30, 60, or 75 minutes; p<0.05).

No significant interaction effects were observed for any segmental bioimpedance variable at any frequency (p > 0.05). However, there were significant group and time effects across most segments and frequencies (p = 0.001–0.048). These effects were generally most pronounced at 50 kHz and 250 kHz and were most consistent for Z and R, followed by Xc and PhA. Results by segment are summarized below. Full consolidated segmental bioimpedance comparisons are presented in Table 3 and time effects are presented in Figure 4.

**Table 3:** Segmental bioimpedance results at 5, 50, and 250kHz for YA and OA.

**Frequency**	**Segment**	**Bioimpedance**	**YA**	**OA**	**Interaction Effect (p-value)**	**Group Effect (p-value)**	**Time Effect (p-value)**
**5 kHz**	Right Arm	Z (Ω)	400.34 ± 16.84	346.33 ± 14.24*	0.467	0.034	0.590
		R (Ω)	400.34 ± 16.84	346.33 ± 14.24*	0.467	0.034	0.590
		Xc (Ω)	15.46 ± 0.92	14.14 ± 0.78	0.423	0.299	0.161
		PhA (°)	2.22 ± 0.13	2.35 ± 0.11	0.466	0.460	0.055
	Left Arm	Z (Ω)	407.15 ± 15.65	339.93 ± 13.23*	0.503	0.008	<0.001
		R (Ω)	406.86 ± 15.64	339.66 ± 13.22*	0.504	0.008	<0.001
		Xc (Ω)	15.21 ± 0.97	13.42 ± 0.82	0.344	0.189	0.002
		PhA (°)	2.14 ± 0.14	2.27 ± 0.12	0.530	0.493	0.071
	Trunk	Z (Ω)	26.91 ± 0.88	24.18 ± 0.75*	0.841	0.040	0.004
		R (Ω)	26.86 ± 0.88	24.12 ± 0.75*	0.865	0.039	0.006
		Xc (Ω)	1.50 ± 0.22	1.52 ± 0.19	0.393	0.935	0.317
		PhA (°)	3.20 ± 0.50	3.62 ± 0.42	0.404	0.534	0.372
	Right Leg	Z (Ω)	312.38 ± 13.69	250.65 ± 11.57*	0.429	0.006	0.014
		R (Ω)	312.06 ± 13.68	250.46 ± 11.56*	0.429	0.006	0.014
		Xc (Ω)	14.05 ± 0.93	9.59 ± 0.78*	0.543	0.004	0.276
		PhA (°)	2.59 ± 0.14	2.17 ± 0.12*	0.692	0.049	0.544
	Left Leg	Z (Ω)	309.24 ± 13.55	254.73 ± 11.46*	0.220	0.012	0.004
		R (Ω)	308.93 ± 13.54	254.53 ± 11.45*	0.219	0.012	0.004
		Xc (Ω)	13.88 ± 0.90	9.91 ± 0.76*	0.647	0.077	0.011
		PhA (°)	2.58 ± 0.12	2.21 ± 0.10*	0.742	0.044	0.027

**50 kHz**	Right Arm	Z (Ω)	358.42 ± 15.56	311.51 ± 13.15*	0.494	0.044	0.676
		R (Ω)	357.00 ± 15.54	310.50 ± 13.13*	0.494	0.045	0.678
		Xc (Ω)	31.81 ± 1.21	25.08 ± 1.02*	0.467	0.022	0.444
		PhA (°)	5.10 ± 0.14	4.63 ± 0.12*	0.433	0.027	0.340
	Left Arm	Z (Ω)	366.47 ± 14.60	307.67 ± 12.34*	0.605	0.012	<0.001
		R (Ω)	365.13 ± 14.59	306.73 ± 12.33*	0.608	0.012	<0.001
		Xc (Ω)	31.31 ± 1.26	23.91 ± 1.06*	0.411	0.001	<0.001
		PhA (°)	4.91 ± 0.18	4.48 ± 0.15*	0.634	0.099	0.171
	Trunk	Z (Ω)	23.55 ± 0.83	21.59 ± 0.70	0.909	0.103	0.055
		R (Ω)	23.36 ± 0.83	21.47 ± 0.71	0.917	0.115	0.066
		Xc (Ω)	2.99 ± 0.15	2.21 ± 0.13*	0.509	0.003	0.002
		PhA (°)	7.31 ± 0.39	5.90 ± 0.33*	0.457	0.021	0.048
	Right Leg	Z (Ω)	276.37 ± 12.17	227.48 ± 10.29*	0.330	0.012	0.006
		R (Ω)	274.85 ± 12.14	226.73 ± 10.26*	0.328	0.013	0.006
		Xc (Ω)	28.50 ± 1.82	18.38 ± 1.54*	0.521	0.002	0.018
		PhA (°)	5.96 ± 0.35	4.61 ± 0.29*	0.451	0.014	0.050
	Left Leg	Z (Ω)	273.45 ± 11.72	231.10 ± 9.91*	0.145	0.020	0.004
		R (Ω)	271.95 ± 11.66	230.35 ± 9.85*	0.143	0.021	0.004
		Xc (Ω)	28.39 ± 1.85	18.45 ± 1.56*	0.485	0.002	0.004
		PhA (°)	5.97 ± 0.30	4.55 ± 0.26*	0.588	0.005	0.018

**250 kHz**	Right Arm	Z (Ω)	323.72 ± 14.28	284.75 ± 12.07	0.468	0.064	0.670
		R (Ω)	322.73 ± 14.25	284.05 ± 12.04	0.470	0.065	0.676
		Xc (Ω)	25.37 ± 1.03	19.90 ± 0.87*	0.314	0.002	0.087
		PhA (°)	4.50 ± 0.10	4.02 ± 0.08*	0.387	0.004	0.048
	Left Arm	Z (Ω)	331.89 ± 13.56	281.75 ± 11.46*	0.624	0.018	<0.001
		R (Ω)	330.87 ± 13.54	281.07 ± 11.44*	0.625	0.018	<0.001
		Xc (Ω)	25.96 ± 1.03	19.55 ± 0.87*	0.611	0.001	0.001
		PhA (°)	4.61 ± 0.16	3.95 ± 0.14*	0.696	0.010	0.013
	Trunk	Z (Ω)	20.15 ± 0.81	19.10 ± 0.69	0.917	0.345	0.154
		R (Ω)	19.90 ± 0.82	18.82 ± 0.70	0.913	0.339	0.156
		Xc (Ω)	3.10 ± 0.24	3.18 ± 0.20	0.334	0.794	0.490
		PhA (°)	8.85 ± 0.82	9.70 ± 0.69	0.297	0.442	0.618
	Right Leg	Z (Ω)	247.83 ± 11.12	209.96 ± 9.40*	0.295	0.026	0.006
		R (Ω)	246.88 ± 11.09	209.47 ± 9.37*	0.294	0.028	0.006
		Xc (Ω)	21.46 ± 1.27	14.17 ± 1.08*	0.523	0.001	0.009
		PhA (°)	2.73 ± 0.27	4.14 ± 0.23*	0.784	0.002	0.066
	Left Leg	Z (Ω)	245.23 ± 10.44	213.34 ± 8.82*	0.136	0.042	0.003
		R (Ω)	244.27 ± 10.38	212.88 ± 8.77*	0.136	0.043	0.004
		Xc (Ω)	21.60 ± 1.40	13.97 ± 1.18*	0.595	0.002	0.001
		PhA (°)	5.04 ± 0.21	3.72 ± 0.18*	0.802	0.001	0.027

YA: Young adult; OA: older adult;

* *Statistically different from YA (p<0.05)*

**Arms:** Group differences for Xc at 50 kHz (p = 0.002, η^2^ = 0.645) and 250 kHz (p = 0.002, η^2^ = 0.622) reflected large effect sizes, while PhA differences at these same frequencies (p = 0.027–0.004, η^2^ = 0.400–0.586) indicated moderate-to-large effects. Time effects were modest, with a small but significant decrease in PhA at 250 kHz (p = 0.048, η^2^ = 0.200 in the right arm; p = 0.013, η^2^ = 0.276 in the left arm).

**Trunk:** Group and time effects were primarily observed at 5–50 kHz. Both Z and R differed between groups at 5 kHz (p = 0.040 and 0.039; η^2^ = 0.359 and 0.361, respectively), while Xc and PhA differed by group at 50 kHz (p = 0.003 and 0.021, η^2^ = 0.613 and 0.430). Time effects were modest, with a significant decrease in both Z and R over time at 5 kHz (p < 0.001; η^2^ = 0.335–0.352) and smaller but significant reductions in both Xc and PhA at 50 kHz (p = 0.002 and 0.048, η^2^ = 0.340 and 0.212, respectively). No time-related changes were observed at 250 kHz (p > 0.05). There were no group or time effects for the trunk at 250 kHz (p > 0.05).

**Legs:** Significant group and time effects were observed for all bioimpedance variables in both legs across all frequencies (p = 0.001–0.049). Across both legs, YA displayed higher bioimpedance values than OA, particularly at higher frequencies (50–250 kHz), where the largest effect sizes were observed for Xc (η^2^ up to 0.64) and PhA (η^2^ up to 0.690). Over time, small-to-moderate declines in all bioimpedance values were observed, most consistently for Z and R (right leg: p = 0.006–0.014, η^2^ = 0.370–0.431; left leg: p = 0.003–0.004, η^2^ = 0.395–0.413). In contrast, smaller, frequency-dependent time effects were observed for Xc and PhA (Xc: p = 0.003–0.018, η^2^ = 0.314–0.437; PhA: p = 0.018–0.050, η^2^ = 0.241–0.264).

### Whole-body fluid

No significant interaction effects were observed for any whole-body fluid variable (p > 0.05). There were no significant group effects for any variable (p = 0.217–0.578, η^2^ = 0.03–0.66), but there were significant time effects for TBW (p = 0.017, η^2^ = 0.279), ICW (p = 0.046, η^2^ = 0.207), and ECW (p = 0.039, η^2^ = 0.336). For the ECW/ICW ratio, a significant group effect was observed (p = 0.001, η^2^ = 0.664), with higher values in OA compared to YA, but there was no significant time effect (p = 0.065, η^2^ = 0.199). Consolidated whole-body fluid comparisons are displayed in Table 4 and Figure 4. Similar results for whole-body fluid were observed in the female-only comparison between YA and OA (Supplementary Table 3).

**Table 4:** Whole-body fluid between YA and OA.

**Fluid**	**YA**	**OA**	**Interaction Effect (p-value)**	**Group Effect (p-value)**	**Time Effect (p-value)**
**TBW (L)**	34.81 ± 2.36	37.40 ± 2.00	0.873	0.421	0.018
**ICW (L)**	21.71 ± 1.45	22.71 ± 1.23	0.843	0.608	0.059
**ECW (L)**	13.10 ± 0.92	14.69 ± 0.78	0.882	0.217	0.002
**ECW/ICW**	0.60 ± 0.01	0.65 ± 0.01*	0.699	0.002	0.025

TBW: total body water; ICW: intracellular water; ECW: extracellular water; ECW/ICW: extracellular-intracellular water ratio

* *Statistically different from YA (p<0.05)*

### Segmental fluid

No significant interaction effects were observed for any segmental fluid variable (p > 0.05). There were no significant group effects for any segment (p = 0.142–0.647, η^2^ = 0.02–0.40), but there were significant time effects. Segmental fluid results are summarized below. Full consolidated segmental fluid comparisons are presented in Table 5, and corresponding comparisons are illustrated in Figure 6.

**Fig.5:**
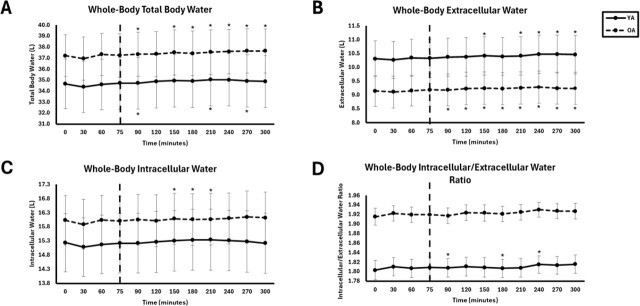
Whole-Body fluid between young adults (YA) and older adults (OA). The EAA challenge was consumed at the 75^th^ minute. *Significantly different from pre-EAA consumption timepoints (0, 30, 60, or 75 minutes; p<0.05).

**Fig.6:**
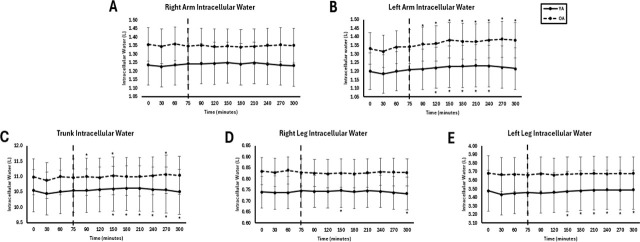
Segmental intracellular water between young adults (YA) and older adults (OA). The EAA challenge was consumed at the 75^th^ minute. *Significantly different from pre-EAA consumption timepoints (0, 30, 60, or 75 minutes; p<0.05).

**Table 5:** Segmental fluid between YA and OA.

**Fluid**	**Segment**	**YA**	**OA**	**Interaction Effect (p-value)**	**Group Effect (p-value)**	**Time Effect (p-value)**
**TBW (L)**	Right arm	1.98±0.19	2.18±0.16	0.632	0.453	0.837
	Left arm	1.95 ± 0.19	2.20 ± 0.16	0.606	0.327	0.003
	Trunk	16.92 ± 1.18	18.11 ± 1.00	0.700	0.460	0.092
	Right leg	5.59 ± 0.42	6.17 ± 0.30	0.708	0.311	0.052
	Left leg	5.58 ± 0.39	6.11 ± 0.31	0.346	0.323	0.017
**ICW (L)**	Right arm	1.24 ± 0.09	1.35 ± 0.10	0.655	0.499	0.830
	Left arm	1.22 ± 0.11	1.36 ± 0.10	0.629	0.369	0.003
	Trunk	10.55 ± 0.72	11.00 ± 0.60	0.651	0.647	0.204
	Right leg	3.48 ± 0.23	3.60 ± 0.25	0.639	0.639	0.045
	Left leg	3.47 ± 0.23	3.51 ± 0.24	0.143	0.540	0.020
**ECW (L)**	Right arm	0.74 ± 0.07	0.83 ± 0.06	0.562	0.386	0.811
	Left arm	0.73 ± 0.07	0.84 ± 0.07	0.586	0.268	0.004
	Trunk	6.37 ± 0.46	7.11 ± 0.35	0.851	0.249	0.023
	Right leg	2.10 ± 0.12	2.44 ± 0.10	0.801	0.142	0.084
	Left leg	2.11 ± 0.12	2.43 ± 0.11	0.440	0.148	0.039
**ECW/ICW**	Right arm	0.60 ± 0.06	0.62 ± 0.05*	0.722	0.002	0.475
	Left arm	0.60 ± 0.04	0.62 ± 0.00*	0.791	0.003	0.488
	Trunk	0.60 ± 0.06	0.65 ± 0.07*	0.923	0.002	0.130
	Right leg	0.60 ± 0.06	0.66 ± 0.06*	0.709	0.003	0.141
	Left leg	0.61 ± 0.06	0.66 ± 0.06*	0.714	0.004	0.102

TBW: total body water; ICW: intracellular water; ECW: extracellular water; ECW/ICW: extracellular-intracellular water ratio

* *Statistically different from YA (p<0.05)*

TBW significantly changed over time in the left arm, right leg, and left leg (p = 0.003–0.017, η^2^ = 0.278–0.396). Similar time effects were observed for ICW in the left arm, right leg, and left leg (p = 0.003–0.045, η^2^ = 0.289–0.385) and for ECW in the trunk (p = 0.023, η^2^ = 0.253) and left leg (p = 0.039, η^2^ = 0.262). The ECW/ICW ratio exhibited significant group effects across all segments (p = 0.002–0.004, η^2^ = 0.581–0.648), with OA consistently displaying higher ratios than YA, indicating a greater extracellular fluid proportion. There were no time effects for ECW/ICW (p = 0.112–0.483, η^2^ = 0.084–0.186).

## Discussion

Raw bioimpedance values offer a quick, non-invasive approach to assess muscle quality (24), nutritional status (13), as well as overall health and disease states (13,25). Results of this exploratory study demonstrated that raw bioelectrical impedance effectively differentiated between younger and older adults as supported by significant group effects for (Z, R, Xc, PhA). This trend was observed particularly in the leg segments at higher frequencies, while post-prandial trends in raw bioimpedance values and fluid shifts suggest MF-BIA is sensitive to postprandial EAA intake. Although full interpretation and generalizability of these results are limited by the small sample size, the study offers novel insight into the potential application of bioimpedance in detecting postprandial physiological responses, warranting further rigorous investigation.

### Whole-body and segmental bioimpedance

In the current study, raw bioimpedance values effectively distinguished between younger and older adults, with consistent group differences observed across both whole-body and segmental measures. The trend of YA > OA, specifically for Xc and PhA, is consistent with previous findings demonstrating that younger individuals typically exhibit higher bioimpedance values (7,12,13), particularly in the legs (26). Leg PhA has been independently associated with walking speed and physical function in aging populations (26). Conversely, the lower Xc and PhA observed in OA are in line with expected age-related declines in muscle quality, cell membrane integrity, and functional capacity (13,27,28). Resistance also followed the trend of YA > OA. However, R is commonly associated with hydration and muscle mass, and therefore is typically lower in healthy, well-hydrated individuals and higher in aging or clinical populations (7,27). Since muscle mass was relatively similar between YA (SMM=26.2 kg) and OA (SMM=27.5 kg), lower resistance in the OA group may be attributed to higher TBW (+2.6 L; p=0.421) and significantly higher ECW/ICW (+0.11 L; p=0.001), compared to the YA group, but further interpretation of this result is required.

Significant time effects were also observed across whole-body and segmental bioimpedance values, with the general trend showing higher values between 0–120 minutes and lower values from 150–300 minutes post-ingestion. This aligns with prior studies reporting decreases in Z and R following mixed meals (29,30) or fluid intake (31) over similar timeframes. These reductions have been attributed to postprandial shifts in fluid compartments, particularly expansion of extracellular water and electrolyte distribution during digestion (30). While the present study observed similar time-dependent trends, interpretation of physiological mechanisms remains speculative given the absence of gold-standard reference methods (e.g., isotope tracers). Theoretically, maximal nutrient uptake typically occurs within 60 minutes postprandially under normal metabolic conditions (32), while deviations from this pattern could suggest altered nutrient handling or metabolic dysfunction (33). From a bioimpedance perspective, that could present as decreasing R reflecting increased intracellular water associated with nutrient uptake by skeletal muscle, with peak effects observed between 60 and 120 minutes. While this pattern was observed in the present study, further research is warranted to fully decipher physiological mechanisms and nutritional sensitivity.

### Whole-body and segmental fluid

All fluid values were higher in OA than YA, however the only significant group effect was for ECW/ICW ratio. This is consistent with prior research that has established there to be general increases in ECW and ECW/ICW with age (34). Counter to expectation, ICW also trended higher in OA, a characteristic typically observed in younger individuals with greater muscle quality and mass (35). However, this result is supported by the bioimpedance results, specifically, the higher R values observed in YA, which may reflect lower hydration status, which was not controlled for in this study.

Time effects for both whole-body and segmental fluid shifts indicated significant changes over the postprandial period, with TBW, ICW, and ECW values generally lower early on and increasing from 180 to 300 minutes. These trends are consistent with bioimpedance time effects, particularly decreases in R over time, which suggest progressive increases in fluid content. Segmental fluid shifts were most apparent in the legs, which also demonstrated the most pronounced time-dependent changes in bioimpedance. The lack of time effects in ECW/ICW complements the bioimpedance results, as PhA and Xc—indicators of membrane integrity and compartmental fluid distribution—exhibited minimal fluctuation in OA over time. Together, the fluid data reinforce previous findings that indicate MF-BIA is sensitive to postprandial fluid shifts (36,37).

## Conclusion

This study highlights the potential of raw bioimpedance values as a non-invasive tool to assess muscle quality and fluid shifts in response to EAA intake. Due to the small sample size, this study should be considered largely exploratory, and results should be interpreted with caution. However, the significant differentiation between younger and older adults in bioimpedance outcomes, particularly in the leg segments, reinforces the utility of bioimpedance for characterizing changes in cellular and skeletal muscle health that occur with aging. Observed post-prandial trends also indicate potential utility for post-prandial bioimpedance sensitivity to acute EAA intake. In addition to conducting a larger scale study, with a more targeted population, under more rigorously controlled conditions, implementation of bioelectrical impedance spectroscopy in future studies, rather than MF-BIA, may provide greater insight into bioimpedance chances specific to the intra- and extracellular spaces (38). Additionally, techniques such as electrical impedance myography may provide more direct insight at the muscle level (39). Collectively, patterns observed in the current study suggest that with further research, development, and validation, bioelectrical impedance outcomes may be sensitive to physiological responses to nutrient intake, which could be used to evaluate valuable information on muscle sensitivity to nutritional intake.

## Supplementary data

**Supplementary Table 1.** Female-Only Demographics

	**YA**	**OA**
**Age (yrs)**	28.5 ± 1.9	73.8 ± 2.1*****
**Height (cm)**	163.3 ± 4.5	163.0 ± 1.8
**Weight (kg)**	76.8 ± 26.8	75.6 ± 8.8
**BMI (kg/m^2^)**	28.7 ± 9.2	28.5 ± 3.7
**SMM (kg)**	24.9 ± 3.4	24.5 ± 0.80
**%BF**	37.1 ± 16.7	39.7 ± 6.1

* Significantly different from YA (p<0.05)

**Supplementary Table 2.** Female-Only Whole-Body Bioimpedance Between YA and OA at 50kHz

**Bioimpedance**	**YA Mean ± SD**	**OA Mean ± SD**	**Interaction (p-value)**	**Group (p-value)**	**Time (p-value)**	**Group Partial eta**	**Time Partial eta**
**R**	681.52 ± 20.04	580.85 ± 20.04*****	0.256	0.012	<0.001	0.678	0.646
**Xc**	64.27 ± 3.53	46.62 ± 3.53*****	0.334	0.012	<0.001	0.676	0.598
**PhA**	5.39 ± 0.22	4.63 ± 0.22	0.127	0.048	0.031	0.505	0.351

* Significantly different from YA (p<0.05)

**Supplemental Table 3.** Female-Only Fluid Between YA and OA

**Fluid**	**YA**	**OA**	**Interaction (p-value)**	**Group (p-value)**	**Time (p-value)**	**Group Partial eta**	**Time Partial eta**
**TBW**	33.09 ± 1.43	33.37 ± 1.43	0.739	0.894	0.157	0.003	0.246
**ICW**	20.59 ± 0.90	20.29 ± 0.90	0.723	0.823	0.322	0.823	0.172
**ECW**	12.50 ± 0.55	13.08 ± 0.55	0.750	0.489	0.026	0.083	0.372
**ECW/ICW**	0.607 ± 0.01	0.645 ± 0.01*****	0.589	0.044	0.025	0.518	0.362

* Significantly different from YA (p<0.05)

## References

[1] Cholewa JM, Dardevet D, Lima-soares F, de Araújo Pessôa K, Oliveira PH, Dos Santos Pinho JR (2017). Dietary proteins and amino acids in the control of the muscle mass during immobilization and aging: role of the MPS response. Amino Acids.

[2] Cruz-Jentoft AJ, Baeyens JP, Bauer JM, Boirie Y, Cederholm T, Landi F (2010). Sarcopenia: European consensus on definition and diagnosis: Report of the European Working Group on Sarcopenia in Older People. Age Ageing.

[3] Marzetti E, Calvani R, Coelho-Júnior HJ, Landi F, Picca A (2024). Mitochondrial Quantity and Quality in Age-Related Sarcopenia. Int J Mol Sci.

[4] Huang DD, Wang SL, Zhuang CL, Zheng BS, Lu JX, Chen FF (2015). Sarcopenia, as defined by low muscle mass, strength and physical performance, predicts complications after surgery for colorectal cancer. Colorectal Dis.

[5] Schaap LA, van Schoor NM, Lips P, Visser M (2018). Associations of Sarcopenia Definitions, and Their Components, With the Incidence of Recurrent Falling and Fractures: The Longitudinal Aging Study Amsterdam. J Gerontol Ser A.

[6] Rubbieri G, Mossello E, Di Bari M (2014). Techniques for the diagnosis of sarcopenia. Clin Cases Miner Bone Metab.

[7] Castizo-Olier J, Irurtia A, Jemni M, Carrasco-Marginet M, Fernández-García R, Rodríguez FA (2018). Bioelectrical impedance vector analysis (BIVA) in sport and exercise: Systematic review and future perspectives. PLOS ONE.

[8] Gudivaka R, Schoeller DA, Kushner RF, Bolt MJ (1999). Single- and multifrequency models for bioelectrical impedance analysis of body water compartments. J Appl Physiol Bethesda Md 1985.

[9] da Silva BR, Orsso CE, Gonzalez MC, Sicchieri JMF, Mialich MS, Jordao AA (2023). Phase angle and cellular health: inflammation and oxidative damage. Rev Endocr Metab Disord.

[10] Cancello R, Brunani A, Brenna E, Soranna D, Bertoli S, Zambon A (2023). Phase angle (PhA) in overweight and obesity: evidence of applicability from diagnosis to weight changes in obesity treatment. Rev Endocr Metab Disord.

[11] Rees AE, Ward LC, Cornish BH, Thomas BJ (1999). Sensitivity of multiple frequency bioelectrical impedance analysis to changes in ion status. Physiol Meas.

[12] Barbosa-Silva MCG, Barros AJ, Wang J, Heymsfield SB, Pierson RN (2005). Bioelectrical impedance analysis: population reference values for phase angle by age and sex2. Am J Clin Nutr.

[13] Norman K, Stobäus N, Pirlich M, Bosy-Westphal A (2012). Bioelectrical phase angle and impedance vector analysis - Clinical relevance and applicability of impedance parameters. Clin Nutr.

[14] Xia X xin, Li C xiang, Xue X xin, Chen Y jun, He F, Guo H rong (2025). Association between phase angle and all-cause mortality in adults aged 18–49 years: NHANES 1999–2004. Sci Rep.

[15] Bosy-Westphal A, Danielzik S, Dörhöfer RP, Later W, Wiese S, Müller MJ (2006). Phase Angle From Bioelectrical Impedance Analysis: Population Reference Values by Age, Sex, and Body Mass Index. J Parenter Enter Nutr.

[16] Di Vincenzo O, Marra M, Di Gregorio A, Pasanisi F, Scalfi L (2021). Bioelectrical impedance analysis (BIA) -derived phase angle in sarcopenia: A systematic review. Clin Nutr Edinb Scotl.

[17] Blue MNM, Tinsley GM, Hirsch KR, Ryan ED, Ng BK, Smith-Ryan AE (2023). Validity of total body water measured by multi-frequency bioelectrical impedance devices in a multi-ethnic sample. Clin Nutr ESPEN.

[18] Yajima T, Yajima K (2023). Ratio of extracellular water to intracellular water and simplified creatinine index as predictors of all-cause mortality for patients receiving hemodialysis. PloS One.

[19] Serra-Prat M, Lorenzo I, Palomera E, Ramírez S, Yébenes JC (2019). Total Body Water and Intracellular Water Relationships with Muscle Strength, Frailty and Functional Performance in an Elderly Population. A Cross-Sectional Study. J Nutr Health Aging.

[20] Silva AM, Wang J, Pierson RN, Wang Z, Heymsfield SB, Sardinha LB (2005). Extracellular water: greater expansion with age in African Americans. J Appl Physiol.

[21] Valensi P, L'Hermite F, Behar A, Sandre-Banon D, Cohen-Boulakia F, Attali JR (2000). Extra cellular water and increase in capillary permeability to albumin in overweight women with swelling syndrome. Int J Obes.

[22] Ziegenfuss T, Lowery L, Lemon P (1998). Acute fluid volume changes in men during three days of creatine supplementation. J Exerc Physiol.

[23] Tipton KD, Gurkin BE, Matin S, Wolfe RR (1999). Nonessential amino acids are not necessary to stimulate net muscle protein synthesis in healthy volunteers. J Nutr Biochem.

[24] Di Vincenzo O, Marra M, Scalfi L (2019). Bioelectrical impedance phase angle in sport: a systematic review. J Int Soc Sports Nutr.

[25] Lukaski HC, Garcia-Almeida JM (2023). Phase angle in applications of bioimpedance in health and disease. Rev Endocr Metab Disord.

[26] Hirano Y, Yamada Y, Matsui Y, Ota S, Arai H (2022). Lower limb muscle quality and phase angle contribute to the reduced walking speed among older adults. Geriatr Gerontol Int.

[27] Buffa R, Floris G, Marini E (2003). Migration of the bioelectrical impedance vector in healthy elderly subjects. Nutr Burbank Los Angel Cty Calif.

[28] Genton L, Norman K, Spoerri A, Pichard C, Karsegard V, Herrmann FR (2017). Bioimpedance-derived phase angle and mortality among older people. Innov Aging.

[29] Slinde F, Rossander-Hulthén L (2001). Bioelectrical impedance: effect of 3 identical meals on diurnal impedance variation and calculation of body composition12. Am J Clin Nutr.

[30] Fogelholm M, Sievänen H, Kukkonen-Harjula K, Oja P, Vuori I, Ellis KJ, Eastman JD (1993). Effects of Meal and Its Electrolytes on Bioelectrical Impedance. Human Body Composition: In Vivo Methods, Models, and Assessment [Internet].

[31] Deurenberg P, Weststrate JA, Paymans I, van der Kooy K (1988). Factors affecting bioelectrical impedance measurements in humans. Eur J Clin Nutr.

[32] Groen BBL, Horstman AM, Hamer HM, Haan M de, Kranenburg J van, Bierau J (2015). Post-Prandial Protein Handling: You Are What You Just Ate. PLOS ONE.

[33] Alyass A, Almgren P, Akerlund M, Dushoff J, Isomaa B, Nilsson P (2015). Modelling of OGTT curve identifies 1 h plasma glucose level as a strong predictor of incident type 2 diabetes: results from two prospective cohorts. Diabetologia.

[34] Park I, Lee JH, Jang DH, Kim J, Hwang BR, Kim S (2020). Assessment of body water distribution in patients with sepsis during fluid resuscitation using multi-frequency direct segmental bioelectrical impedance analysis. Clin Nutr.

[35] Hioka A, Akazawa N, Okawa N, Nagahiro S (2024). Influence of aging on extracellular water-to-total body water ratio in community-dwelling females. Clin Nutr ESPEN.

[36] Hirsch KR, Blue MNM, Smith-Ryan AE (2023). Sensitivity of Raw Bioimpedance Values to Acute Feeding in Healthy Young Adults: Potential Utility for Application. Meas Phys Educ Exerc Sci.

[37] Tinsley GM, Stratton MT, Harty PS, Williams AD, White SJ, Rodriguez C (2022). Influence of acute water ingestion and prolonged standing on raw bioimpedance and subsequent body fluid and composition estimates. J Electr Bioimpedance.

[38] Sinning WE, Morgan AL, Ellis KJ, Eastman JD (1993). The Effects of Body Position on Bioimpedance Spectroscopy. Human Body Composition: In Vivo Methods, Models, and Assessment [Internet].

[39] Sanchez B, Martinsen OG, Freeborn TJ, Furse CM (2021). Electrical impedance myography: A critical review and outlook. Clin Neurophysiol.

